# Ethnically Tibetan women in Nepal with low hemoglobin concentration have better reproductive outcomes

**DOI:** 10.1093/emph/eox008

**Published:** 2017-04-21

**Authors:** Jang Ik Cho, Buddha Basnyat, Choongwon Jeong, Anna Di Rienzo, Geoff Childs, Sienna R. Craig, Jiayang Sun, Cynthia M. Beall

**Affiliations:** 1 Department of Epidemiology and Biostatistics, Case Western Reserve University, School of Medicine, Cleveland, OH 44109, USA; 2 Patan Hospital, Oxford University Clinical Research Unit-Nepal, Kathmandu, Nepal and Centre for Tropical Medicine and Global Health, University of Oxford, Oxford, UK; 3 Department of Human Genetics, University of Chicago, Chicago, IL 60637, USA; 4 Department of Anthropology, Washington University, St. Louis, MO 63130, USA; 5 Department of Anthropology, Dartmouth College, Hanover, NH 03755, USA; 6 Department of Epidemiology and Biostatistics, Case Western Reserve University, School of Medicine, Cleveland, OH 44109, USA; 7 Department of Anthropology, Case Western Reserve University, Cleveland, OH 44106, USA

**Keywords:** adaptation, high altitude, Tibetan, hemoglobin, reproductive success, female fertility

## Abstract

**Background and objectives**: Tibetans have distinctively low hemoglobin concentrations at high altitudes compared with visitors and Andean highlanders. This study hypothesized that natural selection favors an unelevated hemoglobin concentration among Tibetans. It considered nonheritable sociocultural factors affecting reproductive success and tested the hypotheses that a higher percent of oxygen saturation of hemoglobin (indicating less stress) or lower hemoglobin concentration (indicating dampened response) associated with higher lifetime reproductive success.

**Methodology**: We sampled 1006 post-reproductive ethnically Tibetan women residing at 3000–4100 m in Nepal. We collected reproductive histories by interviews in native dialects and noninvasive physiological measurements. Regression analyses selected influential covariates of measures of reproductive success: the numbers of pregnancies, live births and children surviving to age 15.

**Results**: Taking factors such as marriage status, age of first birth and access to health care into account, we found a higher percent of oxygen saturation associated weakly and an unelevated hemoglobin concentration associated strongly with better reproductive success. Women who lost all their pregnancies or all their live births had hemoglobin concentrations significantly higher than the sample mean. Elevated hemoglobin concentration associated with a lower probability a pregnancy progressed to a live birth.

**Conclusions and implications**: These findings are consistent with the hypothesis that unelevated hemoglobin concentration is an adaptation shaped by natural selection resulting in the relatively low hemoglobin concentration of Tibetans compared with visitors and Andean highlanders.

## Teaser

Elevated maternal hemoglobin concentration associated strongly with lower lifetime reproductive success among ethnically Tibetan women at 3000-4100m in Nepal. These findings are consistent with the hypothesis that unelevated hemoglobin concentration is an adaptation shaped by natural selection resulting in low hemoglobin levels among Tibetans compared with visitors and Andean highlanders.

## INTRODUCTION

Adaptations to the environment arise from evolution by natural selection on heritable phenotypes. These adaptations can be difficult to distinguish among many phenotypes distinctive of populations living in specific environments. Difficulties include establishing the heritable basis of a trait that may also have developmental and environmental influences and linking phenotypes with reproductive success. Social, economic, and public health features may contribute to variation in reproductive success, thereby making it challenging to isolate contributions by heritable biological traits. Detecting natural selection on heritable phenotypes by relating them to reproductive success is essential for understanding how adaptations become established in human populations.

High-altitude populations inform understandings of the adaptive process because virtually every physiological system responds to the severe and unavoidable stress of low partial pressure of oxygen (hypoxia) [1]. Two well documented and distinctive heritable phenotypes characterize high-altitude Tibetans.

The first trait is the percent of oxygen saturation of hemoglobin. It falls with increasing altitude, although there is a wide range of oxygen saturation at any altitude. The trait has significant heritability (*h*^2^) [2, 3] and a major gene for percent of oxygen saturation has been inferred among Tibetans [4]. Candidate gene studies reported associations of saturation with oxygen homeostasis loci [5–8]. Relatively high saturation may benefit residents because it increases the oxygen content of arterial blood and somewhat lowers the physiological stress of high-altitude hypoxia. Tibetan women estimated to have the genotypes for higher oxygen saturation had more surviving children at 3900–4200 m [4], suggesting that natural selection favors higher oxygen saturation among Tibetans.

The second trait is hemoglobin concentration. The pathways for the well-known increase in hemoglobin concentration within days of acute exposure to high altitudes have been described [9]. However, this response varies significantly inter-individuals and inter-populations. Some highlanders, including those from the Andes, show the same high hemoglobin concentration phenotype as acutely exposed lowlanders. In contrast, Tibetan highlanders have relatively low hemoglobin concentration at similar altitudes [10]. Tibetans’ lower average hemoglobin concentration relates to higher physical work capacity [11] and probably lowers the risk of thrombosis, Chronic Mountain Sickness or pre-eclampsia.

Polymorphisms in two main genes of the oxygen homeostasis pathway contribute to variation in hemoglobin concentration in Tibetans. The *EGLN1* locus codes for an oxygen sensor and the *EPAS1* locus codes for the alpha subunit of the hypoxia-inducible factor 2 (HIF2) transcription factor that induces dozens of target genes contributing to the homeostatic responses to hypoxic stress. Genomic studies report signals of natural selection at both *EGLN1* and *EPAS1*. *EGLN1* variants are associated with hemoglobin concentration in some studies [12, 13, males only], but not others [14, 15]. *EPAS1* variants are associated with hemoglobin concentration in four studies of Tibetans from different geographic areas [15–18]. Furthermore, the *EPAS1* alleles associating with lower hemoglobin concentration have elevated frequencies among Tibetans [6, 19–25]. The ‘Tibetan’ alleles at *EPAS1* protect against excessively high hemoglobin concentration, chronic mountain sickness, and low birthweight [26, 27]. These findings suggest that Tibetans’ relatively low hemoglobin concentrations are heritable adaptations reflecting a distinctive gene pool shaped by natural selection.

Therefore, the present study aimed to detect and account for nonheritable sociocultural factors affecting reproductive success and to test the hypotheses that an elevated percent of oxygen saturation of hemoglobin or a low hemoglobin concentration can be associated with higher reproductive success (pregnancies, live births, or children surviving to 15 years of age) among highland Tibetan women. The results provided little support for the elevated-oxygen-saturation hypothesis and strong support for the unelevated-hemoglobin-concentration hypothesis. This study links a distinctive oxygen homeostasis phenotype, hemoglobin concentration, to reproductive success in a contemporary Tibetan sample living under the stress of high-altitude hypoxia.

## MATERIALS AND METHODS

### Study populations

**Figure 1.**
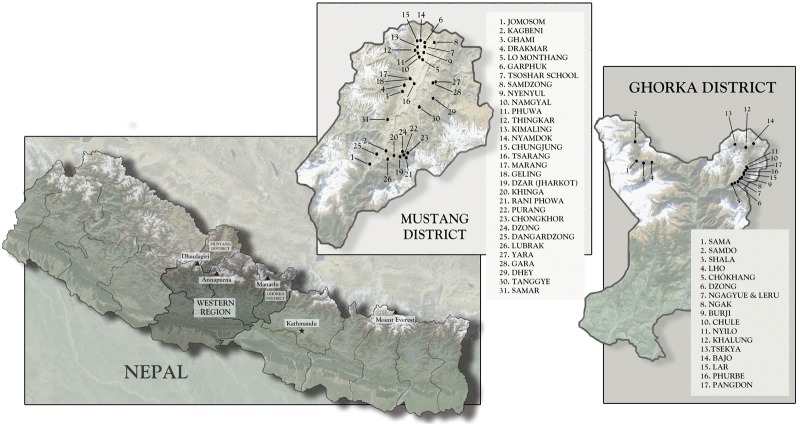
Map of study area (images captured on 11-7-2013 ©Google Image Landsat ©2013 Mapabc.com US Deptartment of State Geographer)

The study communities in Nepal lie on the southern aspects of the Tibetan Plateau. Although they are citizens of Nepal, local people self-identify as ethnically Tibetan: they speak Tibetan dialects, practice forms of religion and social organization akin to those across the Tibetan Plateau, and retain the characteristic agro-pastoral and trading mode of subsistence common among highland Tibetans [28]. Fieldwork took place during the summer of 2012 in (1) the Nubri and Tsum Valleys in Gorkha District and (2) the ethnic Tibetan areas of Upper Mustang and Baragaon in Mustang District (Fig. 1). All regions lie along the border with the Tibet Autonomous Region, China. Nubri and Tsum are contiguous valleys where villages range in altitude from 2090 to 3787 m (6900–12 500’). The inhabitants descend from people who migrated from various nearby areas, including the Tibetan Plateau, beginning in the 14th century or earlier. Nepal incorporated Nubri during the 1850 s [29]. The Tibetan area of Upper Mustang lies along the international border while Baragaon is situated farther south. Villages range in altitude from 2800 to 4200 m (9240–13 860’). Upper Mustang includes the Kingdom of Lo, home of a local hereditary leader whose lineage in the region dates to the 14th century. Upon its founding in the mid-18th century, the nation-state of Nepal incorporated the Kingdom of Lo [30, 31].

The residents of these areas make a living with traditional agriculture (barley, wheat, buckwheat, potatoes, maize) and animal husbandry (yaks and yak-cow crossbreeds, sheep and goats, horses). They also engage in trans-Himalayan trade (timber, medicinal plants), seasonal migration for commodity trade in Indian and Nepali cities, government services, and tourism.

### Study samples

Institutional review boards at Case Western Reserve University, Dartmouth College, the Nepal Health Research Council, Oxford Tropical Research Centre, and Washington University approved the study protocol. Participants provided informed consent.

The reproductive history sample included women who had completed or nearly completed childbearing because they were 40 years of age and older (by Tibetan reckoning, which corresponds to 39 in the western system). All were native to and born at or above 3000 m and had experienced marriage or pregnancy. About 1020 women provided interviews. The final reproductive history sample included 1006 women from 987 households.

The household survey sample included all households in the villages where reproductive history collection took place and provided information on education and wealth. About 1487 household surveys enumerated 8187 people in 63 villages.

### Sample ascertainment

The household survey identified 332 women of appropriate age who were not included in the reproductive survey. 43 of those in Gorkha District and 57 women in Mustang did not meet study selection criteria. Some potentially eligible women were temporarily away from home. Supplementary Table S1 provides details on exclusions. The sample includes over 85% of age-appropriate women in these areas and, therefore, is unlikely to be biased.

## DATA COLLECTION

### Surveys

Research teams of six Nepali research assistants and two of the authors collected data in each study area. Authors GC and SC have been conducting long-term fieldwork in Gorkha and Mustang Districts, respectively. The research assistants were in their twenties, had a secondary education or more, were fluent in Nepali and the local Tibetan dialect and were born in a study village or nearby. The Gorkha District team included two men; the Mustang team included one.

Upon arriving in a village, the team explained to local officials and leaders the purpose of the study and described the data collection process. All parties readily endorsed the research project and provided a list of village households. Teams of two interviewers visited each household in Gorkha District. Similar teams visited households in some villages of Mustang. Other villages made data collection sites available at a temple, school, or community center. The authors rotated among the interview teams to maintain uniform data collection.

A global positioning system measured longitude, latitude, and altitude upon arrival in a village (Garmin eTrex HC series, Garmin International Inc., Olathe, KS). Barometric pressure, temperature and relative humidity were recorded every morning between 6 and 7 am. The median altitude of residence was 3632 m. Supplementary Table S2 lists the residential altitudes for the women in the sample.

The authors trained the interviewers in the protection of human subjects and the collection of reproductive histories, household surveys, and genealogies. Two days of training before data collection included reviewing the informed consent and data collection documents. We discussed the principles of voluntary informed consent, the meaning, and justification of each reproductive history or household survey question, plausible answers and possible misunderstandings. Training also included mock interviews with one another and demonstration interviews for the whole team and with volunteers who were not part of the study. When teams adjusted interview protocols, they were encouraged to add explanatory notes to interview sheets. Training for collecting the biological measurements described below followed the same sequence.

The Tibetan calendar is in daily use in the study communities and enabled researchers, in conversation with study participants, to determine accurate ages and the timing of reproductive history events. This 12-year, repeating cycle of named animal years links inexactly to specific years in the western calendar. A child is considered to be one year old during the year of birth; he or she turns two at the next Tibetan New Year (February or March). For example, a woman with the animal year ‘tiger’ and born in the year 1950 was 63 years of age by Tibetan reckoning in the year 2012 and 62 years of age by western calculation. Interviewers carried a conversion table relating Tibetan ages to western calendar years and used it for age determination and dating events. If a woman reported a chronological age inconsistent with her animal year of birth, then we assumed the latter provided the correct age. In this article, we have converted Tibetan age into western age.

A pregnancy history comprised the core of the reproductive survey. It followed procedures that have been field tested in numerous settings [32]. Questions began with marital history details, then the first pregnancy, the animal year of birth, the outcome (live birth, stillbirth or miscarriage), sex, name, currently alive or dead, age at death and cause of death, if applicable. The reproductive history concluded with information about contraceptive use and, in Mustang District, menopausal status and age at menopause. Women openly discussed issues such as marriage and divorce, miscarriages, stillbirths, infant mortality, and contraception. Other women and sometimes husbands were present during interviews, occasionally clarifying responses or adding details.

Interviewers cross-checked responses for internal consistency and probed as needed for clarifying information. They read the pregnancy history back to the woman for confirmation and asked specifically about pregnancies before the first and after the last on the list to fill in possible omissions. A report of two children born in the same year, such as twins, prompted a follow-up. When a pregnancy history had a gap of three or more years between pregnancies, the interviewer probed for an explanation. About 236 women recalled another pregnancy when asked about specific gaps. The pregnancies added after probing amount to 6.1% of all pregnancies reported. There were no reports of induced or spontaneous abortions. Women probably reported recognized spontaneous abortions as miscarriages. Two women spoke to our research assistants ‘off the record’ about having had an induced abortion. They were uncomfortable reporting this for the record, so reported a miscarriage. The resulting misclassification of 2 out of 129 reported miscarriages may have introduced a small measurement error. The extent of induced abortion underreporting is unknown although it is likely to be very infrequent owing to the limited available health care. In our experience, local Tibetan doctors in the study areas did not make traditional Tibetan abortifacients [33], and women did not mention them. We did not ask about breastfeeding because it was the only option at the time these women had infants.

The household survey recorded the *de jure* population, that is, all individuals born into or married into each household and not officially separated. It included the name, animal year of birth, age, relationship to the household head, education and current whereabouts. The surveyors also asked about land and animal ownership, wage labor, remittances and other sources of household income. In Gorkha District, the main income source was the sale of *Ophio*c*ordyceps sinensis (*a wild fungus in high demand for use in traditional Chinese medicine*)*; in Mustang it was primarily remittances coming from family members living abroad, and from petty seasonal trade in urban Nepal and India. Household economic status was measured using a relative wealth approach. Knowledgeable insiders ranked all village households from one (wealthy) to five (poor) by household assets and other factors [34].

The interviewers returned the completed forms at the end of each day of data collection. The forms were checked for consistency of overlapping information and completeness and then photographed for backup.

### Physiological measurements

Pulse, oxygen saturation of hemoglobin and hemoglobin concentration were measured noninvasively (Masimo Pronto-7 ©, Masimo Corporation, Irvine, CA). The device is accurate to ± 0.99 gm/dL compared to a laboratory reference device (http://masimo.com/pronto-7/index.htm accessed August 1, 2014). Women washed their hands and then sat still with one arm comfortably resting on a flat surface at about heart height, hand resting upon a reusable hand warmer to ensure adequate perfusion. A sensor placed on the forefinger obtained stable readings after a few seconds and then saved a single value for each trait. A reading was not obtained for 47 women, usually because enlarged or misshapen arthritic knuckles prevented the sensor from enclosing the finger. The women without physiological measurements had similar ages, ages at marriage, first and last pregnancy, numbers of pregnancies and live births as the women with those measurements. Therefore, we infer that there was no ascertainment bias in these measurements.

Re-measuring 51 women several months after the interview assessed the reliability and repeatability of the physiological measures. Supplementary Table S3 presents the average pulse, oxygen saturation, and hemoglobin concentration at the first and second measurements for 51 women. Intra-class correlation coefficients (ICC) evaluated the reliability of the repeated measurements [35]. The average difference in hemoglobin concentration evaluated the agreement between the measurements made at different times [36]. Based on the outcomes, the physiological measurements had good to excellent reliability and agreement [37].

### Sample characteristics

**Figure 2.**
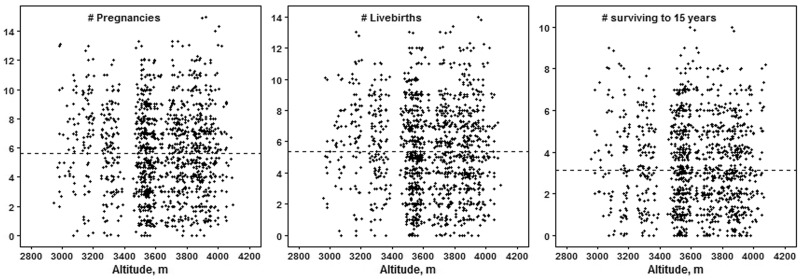
Scatterplot of reproductive success measures with altitude

Three directly obtained *dependent* variables measure reproductive success and offspring survival: numbers of pregnancies, live births and children surviving to the age of 15 years. We chose 15 years because that has been used to indicate reproductive age [38]. Reproductive success varied widely at all residential altitudes (Fig. 2). An average of 5.6 pregnancies resulted in 5.4 live births and four children surviving to the age of 15 (Table 1). Sixty-five percent experienced the death of at least one child before 15 years of age. Numbers of pregnancies and live births correlated very highly (*r* = 0.97) and in turn correlated highly with the number of children surviving to 15 years of age (*r* = 0.72 and 0.74, respectively, all *P* < 0.1E−6). Right censoring occurred in the measure of offspring survival because some women had children born within the past 15 years; the study terminated before these children reached 15 years of age. Therefore, offspring survival analysis included only the 774 women who reported one or more live births born 15 years or more before the study.

**Table 1.** Characteristics of the sample

Continuous variable	*N*		Mean	SD	Min	Max
Pregnancies, #		1006	5.6	2.86	0	15
Live births, #		1006	5.4	2.77	0	14
Children Surviving to the age of 15 years, #		979	4.0	2.14	0	11
Age, years		1006	55	11	39	91
Age at first pregnancy and birth, years		982	24	5	15	46
Age at last pregnancy and birth*, years		982	37	6	19	58
Hemoglobin concentration, gm/dl		959	13.8	1.51	5.2	18.7
O2 Saturation, %		959	87.4	4.5	60	96
Pulse, f/min		958	74	11	49	121
Categorical variable				Count (%)	# Missing (%)	
Current marital status		1 Married		659 (65.51)		
		2 Widowed		252 (25.05)		
		3 Divorced/separate		40 (3.98)		
		4 Never married		55 (5.47)		
Use of contraception		0 Never		682 (67.79)	10 (0.99)	
		1 Past use		147 (14.61)		
		2 Current		167 (16.6)		
District of residence		1 Gorkha		264 (26.24)		
		2 Mustang		742 (73.76)		
Type of marriage		0 Never		55 (5.47)		
		1 Cousin, not polyandrous		72 (7.16)		
		2 Not cousin, is polyandrous		94 (9.34)		
		3 Cousin and polyandrous		16 (1.59)		
		4 Not cousin, not polyandrous		769 (76.44)		

* The single report of 58 years for age at last pregnancy was consistent with the reported pregnancy history. Excluding that report did not change the average age at last pregnancy; it lowered the oldest age to 52 years.

The women averaged 55 years of age (Table 1). Reproductive careers began and ended at average ages of 24 and 37 years with the first and last pregnancies/births. Two percent (*n* = 20) of ever-married women reported no pregnancies. Just under five percent had twin pregnancies, and more than 10% experienced a stillbirth or miscarriage (Supplementary Table S5).

The analysis considered 20 *independent* variables as possible contributors reproductive success. These variables measured social, cultural, economic, and public health factors’ influence on reproductive success independent of the physiological variables of interest. Supplementary Table S4 lists the variables, and Supplementary Table S5 provides descriptive statistics.

The independent variables comprise four groups: direct determinants of exposure to intercourse, direct determinants of susceptibility to conception and successful gestation, indirect determinants of fertility and physiological phenotypes. Direct determinants are variables ‘that must always be operating at some level if reproduction is to occur at all.’ [39 p. 68]. Indirect determinants influence fertility by operating through direct determinants [39–41].

On *direct determinants of exposure to intercourse*, 95% of the women had been married at least once (Table 1). 60% had a single marriage throughout the ages of 25–40 years (categorized as continuously married) and probably had the highest potential exposure to intercourse (Table 1 and Supplementary Table S5).

In regard to other indicators of *direct determinants*, most women had not used contraception (Table 1) [28]. Older women reported that contraception had not been available locally during their childbearing years. Contraception became available in Mustang District during the early to mid-1990s, with the extension of safe motherhood government programs. In the late 1990s, the establishment of several non-governmental organization (NGO)-funded clinics added sources of care. Modern contraception became locally available in Nubri and Tsum sub-districts of Gorkha District in 2009 with the founding of non-governmental-organization-sponsored health posts. This history explains why 38% of Mustang women had used contraception as compared with only 16% of Gorkha District women.

As for *indirect determinants of reproductive success*, roughly 5% had never married, 7% had a cross-cousin marriage, and roughly 11% had a polyandrous marriage (Table 1).

**Figure 3.**
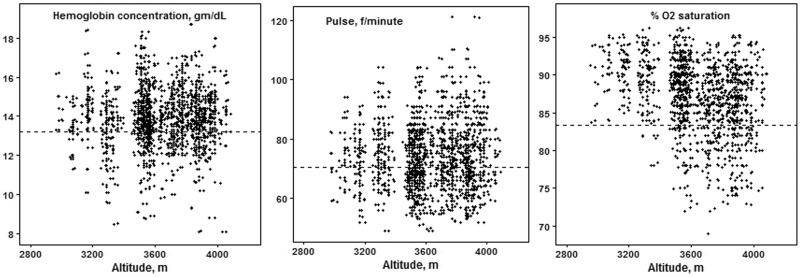
Scatterplot of physiological indicators with altitude. The plot omits two observations (5.2 and 6.1 gm/dl hemoglobin) to improve legibility

*Physiological phenotypes* included pulse, the percent of oxygen saturation of hemoglobin, and hemoglobin concentration (Table 1). No hemoglobin concentration exceeded 19 gm/dL, the threshold for pathologically elevated hemoglobin concentration that is common in Andean high-altitude populations [42]. 11% had hemoglobin concentration below the 12.3 gm/dl used as a cut-off for anemia at low altitudes [43]. Neither hemoglobin concentration nor pulse correlated with altitude. Percent of oxygen saturation of hemoglobin correlated negatively with altitude. The range of variation at each altitude widened to include women with lower saturation at the higher altitudes (Fig. 3). Higher oxygen saturation, a sign of less physiological stress, correlated with lower hemoglobin concentration and lower pulse. These correlations lead us to include pulse in the independent variable lists although we had no *a priori* hypotheses associating pulse with reproductive success.

### Statistical analyses

The analyses excluded the 20 women who had been married yet had never been pregnant; we assumed that 2% of the sample had primary infertility for reasons unrelated to altitude adaptation because the percent is similar to that reported for other samples. For each count outcome, the numbers of pregnancies, live births, and children surviving to 15, we first fit a Poisson regression model with its candidate influential factors. We applied the stepwise selection method to select statistically meaningful factors, and then examined the model fit, including a test of overdispersion, before deciding the `best’ model. If there were an overdispersion, a negative binomial model fit would be adequate; if there were a lack of fit, additional factors, or some transformations of factors would be added, and the model selection and examination steps repeated. In our case, candidates for influential factors considered in all Poisson or negative binomial models consisted of all 20 independent variables representing direct, indirect, and physiological variables, denoted as $\underline{x}$ in Equation (1) below and their possible two-way interactions. To overcome over-parameterization, we included only interactions for which the individual variable itself $x_{i}$ was selected by an initial stepwise selection of 20 variables. These candidate influential factors were connected as a linear predictor inside the parametric model fitting:

(1) $$\eta (x)=\alpha_{0}+\underline{\alpha }*\underline{x'}+\beta *(interactions)$$

where $\underline{\alpha }*\underline{x'}=\alpha_{1}*x_{1}$ + … + $\alpha_{20}*x_{20}$, $\alpha_{i}$’s were estimated and could differ in analyses with different dependent variables, $\alpha_{i}$ not selected in the initial model selection was set to be zero, $\underline{\beta }$ = ($\alpha_{21}$_,_ …) were the coefficients of interaction terms and also estimated using data, and candidate factors $\underline{x}$ = (age, age at first pregnancy and birth, age at last pregnancy and birth, altitude of residence, household relative wealth rank, hemoglobin concentration, percent of oxygen saturation of hemoglobin, pulse, living in natal village, continuously married throughout the ages of 25–40 years, schooling of woman and her husband, household of residence after marriage, marital status, miscarriages, stillbirths, district of residence, sub-district of residence, twin pregnancies, type of marriage, contraceptive use).

To cross validate findings from our parametric model fitting by a Poisson regression analysis, we also conducted a non-parametric procedure using the Classification and Regression Tree (CART). Comparing the cross validated deviances of ‘prune’ and ‘shrink’ trees determined the number of nodes and selected the best CART model. The significant covariates chosen by the best Poisson models were consistent to those found by the corresponding tree models, which confirms the choice of the final Poisson model. The results of the Poisson analysis are presented and examined in detail below while the results of the CART analysis appear in Supplementary Online Figures S1–S3.

The Poisson analyses detected very large effects of variables such as marital status and the relative wealth rank of a woman’s household. For example, women from the wealthiest households averaged 6.5 pregnancies as compared with 4.4 for women from the poorest households. However, neither hemoglobin concentration nor percent of oxygen saturation correlated with ages at first or last pregnancy; mean values did not vary across marital status, marriage type or relative wealth rank categories (data not shown).

Women with more or fewer pregnancies had more insert or fewer opportunities for successful or unsuccessful outcomes regardless of physiological characteristics. Therefore, we addressed the influence of the physiological variables from two additional perspectives by choosing a measure of reproductive success independent of the numbers. We calculated the proportions of successful pregnancies (the number of live births divided by the number of pregnancies) and surviving children (number of children surviving to 15 divided by the number of live births). Binomial regression used both the numerators and denominators as outcomes; that model accommodated all the women in the sample. Also, a linear model in logit (log-odds) scale for the proportions themselves accommodated the subgroups of women who had some pregnancy loss or loss of children. The linear predictors described above in Equation (1) were considered in both the binomial regression and the regression in logit scale to find the important factors contributing to the outcomes in proportions.

The model fit of Poisson regression was examined using residual deviance plots, and the Quasi *R*^2^ and adjusted Quasi *R*^2^ defined below

$$QuasiR^{2}=1-\frac{residualdeviance}{nulldeviance}$$

$$\text{Adjusted}\text{Quasi}R^{2}=1-\frac{residualdeviance/dfresidualdeviance}{nulldeviance/dfnulldeviance}$$

Quasi *R*^2^ and adjusted Quasi *R*^2^ defined here are analogous to the *R*^2^ or adjusted *R*^2^ typically provided with a linear regression fit. However, the Quasi *R*^2^, do not indicate the proportion of variability in the outcome variable explained by the independent variables (Supplemental Table 6). The binomial regression and linear regression on logit scale was examined using likelihood ratio test (LRT) and the p-value of the test is provided to show the goodness-of-fit.

Descriptive tables and text report means and standard deviations or percentages. We adopted a critical value of *P* < 0.05 to indicate statistical significance and *P* < 0.01 to indicate strongly significant.

## RESULTS

At the time of data collection in 2012, the women who comprise our sample reported 5667 pregnancies starting in 1943 and 3986 children who had survived to 15 years of age or older. We consider the influence of social, economic and public health factors as well as physiological characteristics to account for the variation in reproductive success (Tables 2–8).

**Table 2.** Factors associated with the number of pregnancies (*n* = 862, Adjusted Quasi *R*^2^ = 0.739)

Pregnancies	Estimate	Std. Error	*z* value	Pr(>|*z*|) = p
(Intercept)	2.902143	0.613219	4.733	2.22E−06***
Age	−0.0018	0.002741	−0.656	0.51169
Age at 1st pregnancy and birth	−0.11968	0.025865	−4.627	3.71E−06***
Age at last pregnancy and birth	0.018614	0.014658	1.27	0.20413
Pulse (f/min)	−0.00199	0.001313	−1.515	0.12977
# of miscarriages	0.090508	0.027639	3.275	0.00106**
# of stillbirths	0.084306	0.027755	3.038	0.00238**
# of twin pregnancies	0.147806	0.059433	2.487	0.01288*
Mustang district	−0.45168	0.174638	−2.586	0.0097**
Relative wealth rank 2	−0.01746	0.042637	−0.409	0.68221
Relative wealth rank 3	−0.05816	0.044663	−1.302	0.19281
Relative wealth rank 4	−0.10393	0.048402	−2.147	0.03178*
Relative wealth rank 5	−0.1603	0.055952	−2.865	0.00417**
Widowed	−0.01975	0.03741	−0.528	0.59755
Divorced	−0.25629	0.101107	−2.535	0.01125*
Never married	−0.58082	0.112255	−5.174	2.29E−07***
Age:Mustang district	0.006458	0.003166	2.04	0.04137*
Age at 1st pregnancy and birth: age at last pregnancy and birth	0.001479	0.000642	2.304	0.02125*

* *P < 0.05*; *0.05 < P < 0.1*.

** *P < 0.01*.

*** *P < 0.001*.

**Table 3.** Factors associated with the number of live births (*n* = 862, Adjusted Quasi *R*^2^ = 0.717)

	Estimate	Std. Error	*z* value	Pr(>|*z*|)
(Intercept)	3.217333	0.64415	4.995	5.89E−07***
Age	−0.00188	0.002815	−0.667	0.50508
Age at 1st pregnancy and birth	−0.13493	0.027349	−4.934	8.07E−07***
Age at last pregnancy and birth	0.012665	0.015539	0.815	0.41506
Pulse (f/min)	−0.00245	0.001344	−1.821	0.06867
# of miscarriages	−0.0608	0.031469	−1.932	0.05335
# of stillbirths	0.332097	0.203357	1.633	0.10245
# of twin pregnancies	−0.31603	0.354425	−0.892	0.37258
Mustang district	−0.48292	0.179464	−2.691	0.00713**
Relative wealth rank 2	−0.01367	0.043529	−0.314	0.75352
Relative wealth rank 3	−0.06226	0.045565	−1.366	0.17181
Relative wealth rank 4	−0.10884	0.049753	−2.188	0.0287*
Relative wealth rank 5	−0.16521	0.057586	−2.869	0.00412**
Widowed	−0.02227	0.038363	−0.581	0.56153
Divorced	−0.28364	0.105726	−2.683	0.0073**
Never married	−0.55857	0.112477	−4.966	6.83E−07***
Age:Mustang district	0.006937	0.003252	2.133	0.03291*
Age at 1st pregnancy and birth:Age at last pregnancy and birth	0.001822	0.000684	2.663	0.00776**
Age at 1st pregnancy and birth: # of stillbirths	−0.01723	0.008489	−2.029	0.04244*
Age at 1st pregnancy and birth: # of twin pregnancies	0.026109	0.014744	1.771	0.0766

* *P < 0.05; 0.05 < P < 0.1*.

** P < 0.01.

*** *P < 0.001*.

**Table 4.** Factors associated with the probability that a pregnancy resulted in a live birth (*n* = 736, LRT p-value = 1.18E−15)

	Estimate	Std. Error	z value	Pr(>|z|)
(Intercept)	8.85508	1.29001	6.864	6.68E−12***
Age at 1st pregnancy and birth	−0.05032	0.01744	−2.886	0.00391**
Hb gm/dl	−0.23464	0.04734	−4.956	7.20E−07***
Continuously married ages 25–40	0.31118	0.16731	1.86	0.0629
Cousin, not polyandrous marriage	−2.97087	1.05296	−2.821	0.00478**
Not cousin, polyandrous marriage	−1.24268	1.13262	−1.097	0.27257
Cousin, polyandrous marriage	13.22694	988.63183	0.013	0.98933
Not cousin, not polyandrous marriage	−1.74034	1.01557	−1.714	0.08659
Past contraception use	0.40117	0.2077	1.931	0.05342
Current contraception use	−0.42761	0.16917	−2.528	0.01148*
Continuously married ages 25–40: cousin, not polyandrous marriage	1.17485	0.43462	2.703	0.00687**
Continuously married ages 25–40: not cousin, polyandrous marriage	0.51556	0.64402	0.801	0.4234
Continuously married ages 25–40: cousin, polyandrous marriage	−0.26139	1106.94571	0	0.99981

* *P < 0.05; 0.05 < P < 0.1*.

** *P < 0.01*.

*** *P < 0.001*.

**Table 5.** Subgroup of women who had pregnancy loss: factors associated with the log of odds ratio that a pregnancy resulted in a live birth (subgroup with 0 < p < 1, *n* = 163, LRT p-value = 1.69E−9)

	Estimate	Std. Error	*z* value	Pr(>|*z*|)
(Intercept)	0.95794	0.36791	2.604	0.0101*
Age at 1st pregnancy and birth	−0.05974	0.01062	−5.622	8.26E−08***
Age at last pregnancy and birth	0.04496	0.00924	4.865	2.73E−06***
Not cousin, polyandrous marriage	0.5369	0.2143	2.505	0.0132*
Not cousin, not polyandrous marriage	0.13076	0.13838	0.945	0.3461

* *P < 0.05*; *0.05 < P < 0.1*.

** *P < 0.01*.

*** *P < 0.001*.

**Table 6.** Factors associated with the number of live births surviving to 15 years of age (*n* = 541, Adjusted Quasi *R*^2^ = 0.604)

	Estimate	Std. Error	z value	Pr(>|z|)
(Intercept)	−0.67550	1.64E+00	−0.412	0.68064
Age	0.03596	2.48E−02	1.449	0.14732
Age at 1st pregnancy and birth	−0.11510	5.81E−02	−1.983	0.04735*
Age at last pregnancy and birth	0.10290	4.27E−02	2.41	0.01595*
Mustang district	−0.12320	5.69E−01	−0.217	0.82852
Past contraception use	1.23800	5.55E−01	2.23	0.02575*
Current contraception use	0.03306	1.04E+00	0.032	0.97468
Age: age at 1st pregnancy and birth	0.00010	7.62E−04	0.127	0.89863
Age: age at last pregnancy and birth	−0.00102	4.45E−04	−2.286	0.02225*
Age: Mustang district	−0.01044	7.87E−03	−1.327	0.18452
Age at 1st pregnancy and birth: Age at last pregnancy and birth	0.00095	1.33E−03	0.718	0.47255
Age at 1st pregnancy and birth: Mustang district	0.00739	1.36E−02	0.542	0.5879
Age at last pregnancy and birth: Mustang district	0.01426	1.29E−02	1.106	0.26882
Age at last pregnancy and birth: past contraception use	−0.02800	1.48E−02	−1.892	0.05849
Age at last pregnancy and birth: current contraception use	−0.02298	2.41E−02	−0.954	0.34012
Mustang district: current contraception use	0.99020	7.42E−01	1.335	0.18192
Tsum sub district	0.13180	1.08E−01	1.224	0.221
Baragaon sub district	0.19430	7.05E−02	2.757	0.00584**
Tsum sub district: Current contraception use	1.44900	8.55E−01	1.694	0.09031
Baragaon sub district: Past contraception use	−0.01857	1.58E−01	−0.118	0.90623
Baragaon sub district:Current contraception use	−0.10190	1.90E−01	−0.537	0.59118

* *P < 0.05*; *0.05 < P < 0.1*.

** *P < 0.01*.

*** *P < 0.001*.

**Table 7.** Factors associated with the probability that a live birth survived to 15 years (*n* = 736, LRT p-value = 2.2E−16)

	Estimate	Std. error	*z* value	Pr(>|*z*|)
(Intercept)	0.979959	0.383238	2.557	0.01056*
Age	−0.010379	0.005053	−2.054	0.03998*
Pulse (f/min)	0.006803	0.003609	1.885	0.05941
# of miscarriages	−0.113671	0.07897	−1.439	0.15003
# of twin pregnancies	−0.274874	0.159618	−1.722	0.08506
Continuously married ages 25–40	−0.210163	0.08737	−2.405	0.01615*
Past contraception use	0.387793	0.118304	3.278	0.00105**
Current contraception use	0.360245	0.148742	2.422	0.01544*
Husband moved to wife’s home	−0.18616	0.116749	−1.595	0.11082
Couple made a new household	−0.234336	0.13041	−1.797	0.07235
Mustang district	0.028055	0.108671	0.258	0.79628
Tsum sub district	0.44591	0.14555	3.064	2.19E−03**
Baragaon sub district	0.850447	0.143821	5.913	3.35E−09***

* *P* < 0.05; 0.05 <* P* < 0.1.

** *P* < 0.01.

*** *P* < 0.001.

**Table 8.** Subgroup of women who had some child loss: factors associated with the log of odds ratio that a live birth survived to 15 years of age (subgroup 0 < p < 1, *n* = 448, LRT p-value = 1.01E−6)

	Estimate	Std. Error	*z* value	Pr(>|*z*|)
(Intercept)	−0.42881	0.70430	−0.60900	0.54295
Age at 1st pregnancy and birth	−0.02942	0.01015	−2.90000	0.00392**
Age at last pregnancy and birth	0.01908	0.00709	2.69100	0.00741**
Altitude of residence	0.00031	0.00018	1.69700	0.09049
Living in natal village	−0.12805	0.07320	−1.74900	0.08093
Relative wealth rank 2	−0.10943	0.10803	−1.01300	0.31162
Relative wealth rank 3	−0.07062	0.11227	−0.62900	0.52965
Relative wealth rank 4	−0.23412	0.11831	−1.97900	0.04846*
Relative wealth rank 5	−0.31428	0.13172	−2.38600	0.01746*
Past contraception use	0.22539	0.09715	2.32000	0.0208*
Current contraception use	−0.18799	0.11809	−1.59200	0.11214
Mustang district	−0.03400	0.11776	−0.28900	0.77294
Tsum sub district	0.21820	0.13650	1.59800	0.11066
Baragaon sub district	0.48304	0.13611	3.54900	0.00043***

* *P* < 0.05; 0.05 < *P* < 0.1.

** *P* < 0.01.

*** *P* < 0.001.

## INDIRECT INFLUENCES ON FERTILITY AND OFFSPRING SURVIVAL

Indirect influences included relative wealth rank and residence district or sub-district. Relative wealth ranks 1 (highest) to 5 (lowest) showed a gradient of effect sizes from the most to the fewest pregnancies and live births (Tables 2, 3 and 8). Residence in Mustang District was associated with fewer live births and pregnancies. Women in the Baragaon sub-district of Mustang had more children surviving to age 15 (Table 6), likely reflecting the longer history of nearby medical care, including childhood vaccinations.

### Direct influences on fertility and offspring survival

#### Exposure to the risk of intercourse

We used marital status and marital type to assess the risk of intercourse. Never-married women had the lowest reproductive success: an average of 1.9 ± 1.43 (*n* = 55) pregnancies compared with 6.0 ± 2.75 (*n* = 646) among those married at the time of the survey. However, never-married women had the same probability of a successful pregnancy outcome and child survival to 15 years of age as those of other marital statuses. Cross-cousin marriage (to the son of mother's brother or father's sister) to one husband associated with a lower probability that a pregnancy became a live birth (Table 5). The effect was smaller among women who were continuously married to cross-cousins.

#### Risk of conception

Contraceptive use did not associate with pregnancies or live births. It related to more children surviving to the age of 15 and with a higher probability that a live birth survived to 15 (Tables 6 and 7). 87% of the contraceptive users wanted to stop pregnancy, explaining that they had enough children (many also mentioned the cost of raising children and concerns for their own health) while only 7% reported using contraception to space births.

#### Successful gestation

Twin pregnancies, miscarriages, and stillbirths related to more pregnancies. The lack of association with live births or surviving children suggests that the additional pregnancies simply replaced the pre-natal losses (Tables 2, 3 and 6). Shorter intervals between pregnancies partly accounted for the direct relationship. If a first and second pregnancy both ended in live births, the modal interval between pregnancies/births was two years. However, when a first pregnancy ended in a miscarriage or stillbirth, the modal interval between the first and second pregnancy was one year. The intervals between births 2 and 3 or 3 and 4 showed the same acceleration of pregnancies following a prenatal loss.

#### Life history

Age at first and last pregnancy both related strongly to reproductive success. Older age associated with a lower probability that a live birth survived to 15 years of age (Table 7). A later first birth also conferred a strong reproductive disadvantage measured as a lower probability that a pregnancy progressed to a live birth, fewer pregnancies, live births, and surviving children (Tables 2–6).

On the other hand, a later last birth conferred a reproductive advantage measured as more live births and more children surviving to adulthood (Tables 3 and 6). Higher rates at which a pregnancy became a live birth or a live birth survived to 15 years partly explained this advantage (Tables 5 and 8). Later last births also partly accounted for the association of miscarriages and stillbirths with more pregnancies. Among those reporting zero, one or two miscarriages, last pregnancies occurred at 37 ± 6, 38 ± 5.9 and 40 ± 5.2 years of age, respectively (one-way ANOVA, *F* (2, 977), *P* = 0.001). The combination of shorter time between pregnancies and later end of reproduction resulted in more pregnancies overall.

### Physiological phenotypes

We hypothesized that higher percent of oxygen saturation of hemoglobin and lower hemoglobin concentration associated with reproductive success. Percent of oxygen saturation did not associate with these outcome measures. Higher hemoglobin concentration associated strongly with a lower probability a pregnancy progressed to a live birth (Table 4).

Taking a different analytical approach by comparing women from the extremes of reproductive failure and success provides additional support for the regression analysis finding that hemoglobin concentration influenced reproductive success. Six women reported that none of their pregnancies became live births, 208 reported pregnancy loss and 720 reported that all their pregnancies became live births. Percent of oxygen saturation and pulse did not vary among those three groups. However, hemoglobin concentration lowered with greater reproductive success: from an average of 14.7 ± 1.5, to 14.2 ± 1.3, and to 13.7± 1.5 gm/dl among women with no, intermediate and complete success in converting pregnancies to live births (one-way ANOVA *F*(2, 860) = 6.153, *P* < 0.01).

Continuing with the approach of comparing extremes, 26 women reported that none of their live births survived to 15 years, 539 reported some loss and 342 reported that all their live births survived 15 years. Pulse did not differ among the three groups. However, the average percent of oxygen saturation was 1.7% higher in the group with complete child survival, and the average hemoglobin concentration was 0.9 gm/dl lower as compared to the group reporting no offspring survival. The average oxygen saturation increased with better reproductive success: from 84.4 ± 5.9, 87.2 ± 4.6–87.9 ± 4.3% (one-way ANOVA *F* (2, 860 = 7.228, *P* < 0.001). The average hemoglobin concentration decreased with reproductive success: from 14.7 ± 1.6, to 14.0 ± 1.4, and to 13.6 ± 1.6 gm/dl (one-way ANOVA *F* (2,860 = 6.153, *P* < 0.01). The 26 women with no surviving children were another tiny fraction of the sample, yet their characteristics contribute to understanding those among whom selection may be strongest.

Importantly, the two sub-groups with the most successful reproductive outcomes identify the hemoglobin concentration associated with most favorable reproductive outcomes. It was 13.6–13.7 gm/dl, slightly lower than the 13.8 *±* 1.5 gm/dl sample average.

## CONCLUSIONS AND IMPLICATIONS

Women with average hemoglobin concentration had a higher probability that pregnancies progressed to live births compared with those having elevated hemoglobin concentration. Furthermore, women whose pregnancies all ended in live births or whose children all survived had hemoglobin concentrations near the sample mean. The consistent pattern supports the hypothesis that natural selection is acting against elevated hemoglobin concentration, or something closely correlated with it, among high-altitude resident Tibetan women.

As for the other physiological measurements, higher pulse related only marginally to a higher probability of child survival. Women with live births but no children surviving to 15 years of age had significantly lower oxygen saturation than others. That result was broadly consistent with a different study of the reproductive success of younger (20–59 years of age) Tibetan women at 3800–4200 m. It found that women with inferred genotypes for lower oxygen saturation had fewer living children [4]. The agreement of these two studies with different designs, age ranges, and analyses both finding that higher oxygen saturation associated with higher offspring survival bolsters the present study’s weak support for the hypothesis that natural selection favors higher oxygen saturation.

Indirect and direct factors influenced reproductive success. Importantly, physiological phenotypes did not vary with indirect or direct factors such as relative wealth or marital status. Based on the estimates of effect sizes provided by the regression equations, the largest reproductive disadvantages accrued to women who never married or had a relatively late first birth. Based on probability differences, residence in Baragaon and a continuous marriage favored reproductive success. This unsurprising finding is well known among demographers [39, 41] and should be a crucial methodological consideration when designing studies of natural selection in humans.

Limitations of this study include relying on retrospective data collected at one interview and a lack of vital records or other documentation of demographic events. Recall of events from decades earlier may have been incomplete. However, women 70 years of age and older reported as many events as the total sample. Those 104 women reported an average of 5.8 pregnancies and 3.7 children surviving to reproductive age; 8% recalled miscarriages, and 13% reported stillbirths. Those values are essentially the same as those in the total sample (Table 1, Supplementary Online Table S5). Any under-reporting appears to be unrelated to age. Another indicator of thorough recall and reporting is rates of stillbirths and miscarriages. The rate of reported stillbirths (27.4/1000 pregnancies) in this study is very similar to the rate of 28.9/1000 in a multidisciplinary, 4-year prospective study in seven low-resource countries [44]. The sex-ratio at birth was 1.06 (#males/#females) indicating no female shortage. Thus, we conclude that the women accurately recalled their reproductive histories.

A methodological limitation arises from the technical specifications of the Masimo-Pronto-7© used to measure hemoglobin concentration noninvasively. The device measures to ± 0.99 gm/dl accuracy of the true value provided by a reference machine. The range around the true value introduces measurement noise. On average, there is little difference between values obtained using this noninvasive device and a reference laboratory machine [45]. We chose to accept this limitation to remove any barriers to participation possibly posed by blood draws that would have provided samples for more accurate results.

The extent to which physiological phenotypes collected post-reproductively reflect those during the reproductive period is unknown. A small subset of 27 women from Baragaon participated in a 1981 study by one of the authors [46, 47]. Hemoglobin or pulse measured in 1981 did not correlate with that in 2012, perhaps due to different equipment; maybe these phenotypes are not canalized throughout adulthood, are affected by undetected disease, or perhaps they correlate with another trait under selection. Also, the extent of the phenotypic variation attributable to genetic differences is not known for this sample.

This cross-sectional study cannot directly evaluate whether the association of unelevated hemoglobin concentration with better reproductive success reflects a benefit, as hypothesized, or a cost in the form of maternal depletion owing to multiple pregnancies [48, 49]. Two percent of the 49 women with 10 or more pregnancies were anemic as compared with 6% of the 68 women with a single pregnancy [50]. That is the opposite of expectation if maternal depletion accounted for the association detected here.

As for strengths, three authors with extensive experience conducting reproductive history surveys in Tibetan societies designed the survey and its administration to collect the best quality data in these circumstances [32]. Interviewers were fluent in the local dialects of Tibetan, they used the Tibetan calendar system, and they followed up on potential omissions or errors. Many study participants were already familiar and comfortable with members of the interviewing team. Other strengths of the study include the large sample size, the very high ascertainment of eligible women, the wide age range, multiple data collection sites and the variety of indirect and direct determinants of fertility considered. The sample of 1006 was large enough to detect a range of effect sizes and included a wide scope of realistic life circumstances. The two districts and sub-districts differed in the timing of reproductive history events, income sources, and public health infrastructure. The nearly complete ascertainment avoided sample bias, for example toward more or less fertile women. Overall, the sample selection reduced the chance that a random unknown confounder biased the results. Another strength was quantification of the exposure to the environmental agent of natural selection—high-altitude hypoxia—by including altitude of birth and of current residence as independent variables.

Published census data along with a previous analysis of fertility in Gorkha District led to the expectation of very low fertility after the age of 40. Our findings agreed. Maternal age was 40 years or older for just 381 births (7%) of the 5,378 live births reported altogether. Only nine women ranging from 39 to 51 years of age (0.01% of the sample) were pregnant at the time of the interview. Thus the reported reproductive histories measure lifetime reproductive success for nearly all the sample.

These findings identified a public health concern arising from the cultural practice of cross-cousin marriage (7.4% of the women), which lowered the probability a pregnancy progressed to a successful live birth. The cost was highest among short cross-cousin marriages, intermediate among stable cross-cousin marriages and did not occur among polyandrous cross-cousin marriages (*n* = 15, all their pregnancies became live births).

These results suggest that natural selection in Tibetan populations resulted in dampening the sustained elevation of hemoglobin concentration characteristic of the acclimatization response to visitors and Andean highlanders. The outcome was un-elevated or little-elevated hemoglobin levels compared with healthy low-altitude samples [50]. The phenomenon of selection favoring phenotypes similar to non-stressed populations is known as cryptic adaptive evolution [51].

A prenatal cost of elevated hemoglobin concentration has been reported previously at high and low altitudes. Elevated hemoglobin concentration during gestation relates to poorer pregnancy outcomes [52–57]. For instance, the absence of hemodilution during pregnancy may double the risk of stillbirths [58].

In summary, this study of ethnically Tibetan women residing at 3000-4100 m in Nepal who have completed reproduction took into account sociocultural and public influences on fertility to detect physiological associations. The results provide little support for the hypothesis that relatively high oxygen saturation is an adaptive phenotype**.** The results strongly support the hypothesis that unelevated hemoglobin concentration (within the normal sea level range), or a closely correlated trait, is an adaptive phenotype in the Tibetan high-altitude population.

## SIGNIFICANCE

A crucial test of the hypothesis of natural selection involves linking the range of variation of phenotypes to fertility and offspring mortality patterns. To date, linking genotypes, physiological phenotypes, and reproduction is uncommon [59–61]. To establish such a link, this study took into account nonheritable social, cultural, and public health covariates whose effects may fluctuate from positive to negative during a woman’s lifetime or between generations. The study added to the classic measures of reproductive success—counts of reproductive events. Reproductive success outcomes measured as probabilities and rates incorporated both sets of variables for the entire sample. Although women had sociocultural characteristics associated with fewer pregnancies, the physiological phenotypes of pulse, oxygen saturation, and hemoglobin concentration were distributed throughout the sample regardless of the social, cultural, and public health covariates.

An important review on measuring natural selection in contemporary populations noted that, ‘The major unresolved issues are how to deal with cultural evolution and gene-culture evolution.’ [62 p. 618]. Our study illustrates that one effective way to deal with "culture" is to measure it systematically. The classic demographic model of indirect and direct determinants of fertility provides a structured approach to such measurements than can be tailored appropriately using ethnographic understanding of the sample. The institution of Tibetan fraternal polyandry is relevant for our sample, while education is not, for instance. Existing studies of natural selection in contemporary populations take into account a few ‘confounders’ such as education, date and place of birth or religion [e.g. 60, 63, 64–67]. Two considered socioeconomic status [68, 69]. Interestingly, status as ‘ever married’ or ‘currently in a heterosexual relationship’ was considered in two studies [69, 70], while none of these mentioned accounting for marital status.

Study samples will inevitably have variation in indirect and direct determinants of fertility. Measuring and accounting for them can change estimates of the relative fitness of phenotypes. A comparison of women in our sample with average hemoglobin concentration and those with hemoglobin one standard above the mean illustrates this point. Consider two women of average age at first pregnancy, continuously married to one man who was not a cross cousin, and who never used contraception. 95.1% of the pregnancies became live births for the hypothetical woman with average hemoglobin levels as compared with 93.2% for the woman with elevated hemoglobin (a 1.9% difference). If we allow cousin marriage to two women with the same set of characteristics, then 76.2% of the pregnancies became live births for the woman with average hemoglobin level as compared with 69.3% for the woman with elevated hemoglobin (a 6.9% difference). That is, the cost of elevated hemoglobin varied according to that indirect determinant, implying that the strength of natural selection can vary depending on variation in such non-heritable traits.

Natural selection ‘in the wild’ is generally weak, as discovered by a meta-analysis of published studies of non-humans that found that most estimates of directional selection gradients were between −1 and 1 [68–70]. Our estimate for hemoglobin concentration was consistent with a value −0.23 (Table 4). Our study made the assumption that natural selection is acting on hemoglobin concentration. While hemoglobin concentration was chosen based on the scientific understanding described in the introduction, we cannot exclude the possibility that an unmeasured trait correlated with hemoglobin concentration is the target of selection.

In summary, these results add to the body of evidence that phenotypic selection acting over one or two generations is weak, yet detectable. This study advances our understanding of natural selection among high-altitude Tibetans by linking the heritable phenotype of unelevated hemoglobin concentration, or a correlated trait, to greater reproductive success, consistent with the hypothesis of an adaptation shaped by natural selection.

## Supplementary Data

Supplementary data is available at *EMPH* online.

## Supplementary Material

Supplementary Data

## References

[1] West JB, Schoene RB, Milledge JS. 2007 High Altitude Medicine and Physiology. London: Hodder Arnold.

[2] Beall CM, Strohl KP, Blangero J Quantitative genetic analysis of arterial oxygen saturation in Tibetan highlanders. Hum Biol 1997;69:597–604. 9299881

[3] Beall CM, Almasy LA, Blangero J Percent of oxygen saturation of arterial hemoglobin among Bolivian Aymara at 3,900-4,000 m. Am J Phys Anthropol 1999;108:41–51. 9915300 10.1002/(SICI)1096-8644(199901)108:1<41::AID-AJPA2>3.0.CO;2-K

[4] Beall CM, Song K, Elston RC Higher offspring survival among Tibetan women with high oxygen saturation genotypes residing at 4,000 m. Proc Natl Acad Sci USA 2004;101:14300–4. 15353580 10.1073/pnas.0405949101 PMC521103

[5] Mishra A, Mohammad G, Thinlas T EGLN1 variants influence expression and SaO2 levels to associate with high-altitude pulmonary oedema and adaptation. Clin Sci 2013;124:479–89. 23130672 10.1042/CS20120371

[6] Buroker NE, Ning XH, Zhou ZN EPAS1 and EGLN1 associations with high altitude sickness in Han and Tibetan Chinese at the Qinghai-Tibetan Plateau. Blood Cells Mol Dis 2012;49:67–73. 22595196 10.1016/j.bcmd.2012.04.004

[7] Takeda N, Maemura K, Imai Y Endothelial PAS domain protein 1 gene promotes angiogenesis through the transactivation of both vascular endothelial growth factor and its receptor, Flt-1. Circ Res 2004;95:146–53. 15192019 10.1161/01.RES.0000134920.10128.b4

[8] Buroker NE, Ning XH, Zhou ZN VEGFA SNPs and transcriptional factor binding sites associated with high altitude sickness in Han and Tibetan Chinese at the Qinghai-Tibetan Plateau. J Phys Sci 2013;63:183–93. 10.1007/s12576-013-0257-8 PMC10717492 23553563

[9] Semenza GL. Involvement of oxygen-sensing pathways in physiologic and pathologic erythropoiesis. Blood 2009;114:2015. 19494350 10.1182/blood-2009-05-189985

[10] Beall CM. Andean, Tibetan, and Ethiopian patterns of adaptations to high-altitude hypoxia. Integ Comp Biol 2006;46:18–24. 10.1093/icb/icj004 21672719

[11] Simonson TS, Wei G, Wagner HE Low haemoglobin concentration in Tibetan males is associated with greater high-altitude exercise capacity. J Physiol 2015;593:3207–18. 25988759 10.1113/JP270518 PMC4532538

[12] Simonson TS, Yang Y, Huff CD Genetic evidence for high-altitude adaptation in Tibet. Science 2010;329:72–5. 20466884 10.1126/science.1189406

[13] Xiang K, Ouzhuluobu Peng Y Identification of a Tibetan-specific mutation in the hypoxic gene EGLN1 and its contribution to high-altitude adaptation. Mol Biol Evol 2013;30:1889–98. 23666208 10.1093/molbev/mst090

[14] Wuren T, Simonson TS, Qin G Shared and unique signals of high-altitude adaptation in geographically distinct Tibetan populations. PLoS One 2014; 9:e88252. 24642866 10.1371/journal.pone.0088252 PMC3958363

[15] Jeong C, Alkorta-Aranburu G, Basnyat B Admixture facilitates genetic adaptations to high altitude in Tibet. Nat Commun 2014;5:1–7. 10.1038/ncomms4281 PMC4643256 24513612

[16] Petousi N, Croft QP, Cavalleri GL Tibetans living at sea level have a hyporesponsive hypoxia-inducible factor (HIF) system and blunted physiological responses to hypoxia. J Appl Physiol 2013. 10.1152/japplphysiol.00535.2013 PMC3972739 24030663

[17] Beall CM, Cavalleri GL, Deng L Natural selection on EPAS1 (HIF2alpha) associated with low hemoglobin concentration in Tibetan highlanders. Proc Natl Acad Sci 2010;107:11459–64. 20534544 10.1073/pnas.1002443107 PMC2895075

[18] Yi X, Liang Y, Huerta-Sanchez E Sequencing of 50 human exomes reveals adaptation to high altitude. Science (New York, N.Y.) 2010;329:75–8. 10.1126/science.1190371 PMC3711608 20595611

[19] Hanaoka M, Droma Y, Basnyat B Genetic variants in EPAS1 contribute to adaptation to high-altitude hypoxia in Sherpas. PLoS One 2012;7:e50566. 23227185 10.1371/journal.pone.0050566 PMC3515610

[20] Bigham A, Bauchet M, Pinto D Identifying signatures of natural selection inTibetan and Andean populations using dense genome scan data. PLoS Genet 2010;6:e1001116. 20838600 10.1371/journal.pgen.1001116 PMC2936536

[21] Peng Y, Yang Z, Zhang H Genetic variations in Tibetan populations and high altitude adaptation at the Himalayas. Mol Biol Evol 2011;28:1075–81. 21030426 10.1093/molbev/msq290

[22] Xu S, Li S, Yang Y A genome-wide search for signals of high altitude adaptation in Tibetans. Mol Biol Evol 2011;28:1003–11. 20961960 10.1093/molbev/msq277

[23] Wang B, Zhang YB, Zhang F On the origin of Tibetans and their genetic basis in adapting high-altitude environments. PLoS One 2011;6. 10.1371/journal.pone.0017002 PMC3046130 21386899

[24] Yang YZ, Wang YP, Qi YJ Endothelial PAS domain protein 1 Chr2:46441523(hg18) polymorphism is associated with susceptibility to high altitude pulmonary edema in Han Chinese. Wilderness Environ Med 2013;24:315–20. 24041620 10.1016/j.wem.2013.05.006

[25] Lorenzo FR, Huff C, Myllymaki M A genetic mechanism for Tibetan high-altitude adaptation. Nat Genet 2014;46:951–6. 25129147 10.1038/ng.3067 PMC4473257

[26] Xu XH, Huang XW, Qun L Two functional loci in the promoter of EPAS1 gene involved in high-altitude adaptation of Tibetans. Sci Rep 2014;4:7465. 25501874 10.1038/srep07465 PMC4264014

[27] Xu J, Yang YZ, Tang F EPAS1 gene polymorphisms are associated with high altitude polycythemia in Tibetans at the Qinghai-Tibetan Plateau. Wilderness Environ Med 2015. 10.1016/j.wem.2015.01.002 25792003

[28] Craig SR, Childs G, Beall CM. Closing the womb door: contraception use and fertility transition among culturally Tibetan women in Highland Nepal. Matern Child Health J 2016. 10.1007/s10995-016-2017-x 27167869

[29] Childs G. 2004 Tibetan Diary: From Birth to Death and Beyond in a Himalayan Valley of Nepal. Berkeley: University of California Press.

[30] Dhungel R. 2002 The Kingdom of Lo (Mustang): A Historical Study. Kathmandu: Tashi Gephel Foundation.

[31] Ramble C. 2008 The Navel of the Demoness: Tibetan Buddhism and Civil Religion in Highland Nepal. Oxford: Oxford University Press.

[32] Beall CM, Leslie PW. Collecting women's reproductive histories. Am J Hum Biol 2014;26:577–89. 24665016 10.1002/ajhb.22543 PMC6679975

[33] Adams V, Miller S, Craig S Informed Consent in cross-cultural perspective: clinical research in the Tibetan Autonomous Region, PRC. Cult Med Psychiatry 2007;31:445–72. 17968637 10.1007/s11013-007-9070-2

[34] Childs G. Perceptions of relative wealth in a Tibetan Community. Tibet J 2001;26:26–38.

[35] Shrout PE, Fleiss JL. Intraclass correlations: uses in assessing rater reliability. Psychol Bull 1979;86:420–8. 18839484 10.1037//0033-2909.86.2.420

[36] Bland JM, Altman DG. Statistical methods for assessing agreement between two methods of clinical measurement. Lancet 1986;1:307–10. 2868172

[37] Cicchetti DV. Guidelines, criteria, and rules of thumb for evaluating normed and standardized assessment instruments in psychology. Psychol Assess 1994;6:284–90.

[38] Strassmann BI, Gillespie B. How to measure reproductive success?. Am J Hum Biol 2003;15:361–9. 12704712 10.1002/ajhb.10154

[39] Wood JW. 1994 Dynamics of Human Reproduction: Biology, Biometry, Demography. Hawthorne, NY: Aldine de Gruyter.

[40] Bongaarts J. A framework for analyzing the proximate determinants of fertility. Popul Dev Rev 1978;4:105–32.

[41] Bongaarts J, Potter RG. 1983 Fertility, Biology, and Behavior: An Analysis of the Proximate Determinants. New York: Academic Press.

[42] Leon-Velarde F, Maggiorini M, Reeves JT Consensus statement on chronic and subacute high altitude diseases. High Alt Med Biol 2005;6:147. 16060849 10.1089/ham.2005.6.147

[43] Looker AC, Gunter EW, Johnson CL. Methods to assess iron status in various NHANES surveys. Nutr Rev 1995;53:246–54. 8577407 10.1111/j.1753-4887.1995.tb05481.x

[44] McClure EM, Saleem S, Goudar SS Stillbirth rates in low-middle income countries 2010 - 2013: a population-based, multi-country study from the Global Network. Reprod Health 2015; 12 (Suppl. 2):S7. 10.1186/1742-4755-12-S2-S7 PMC4464024 26063292

[45] Vyas KJ, Danz D, Gilman RH Noninvasive assessment of excessive erythrocytosis as a screening method for chronic mountain sickness at high altitude. High Alt Med Biol 2015;16:162–8. 25973777 10.1089/ham.2015.0026 PMC4490741

[46] Beall CM. Age at menopause and menarche in a high altitude Himalayan Population. 1983; 10:365–70. 10.1080/03014468300006531 6614862

[47] Beall CM, Reichsman AB. Hemoglobin levels in a himalayan high altitude population. Am J Phys Anthr 1984;63:301–6. 10.1002/ajpa.1330630306 6731601

[48] Gurven M, Costa M, Ben T Health costs of reproduction are minimal despite high fertility, mortality and subsistence lifestyle. Scientific Rep 2016;6:30056. 10.1038/srep30056 PMC4951795 27436412

[49] Tracer DP. Somatic versus reproductive energy allocation in Papua New Guinea: life history theory and public health policy. Am J Hum Biol 2002;14:621–6. 12203816 10.1002/ajhb.10073

[50] Looker AC, Dallman PR, Carroll MD Prevalence of iron deficiency in the United States. J Am Med Assoc 1997;277:973–6. 10.1001/jama.1997.03540360041028 9091669

[51] Storz JF, Scott GR, Cheviron ZA. Phenotypic plasticity and genetic adaptation to high-altitude hypoxia in vertebrates. J Exp Biol 2010;213(Pt 24):4125–36. 21112992 10.1242/jeb.048181 PMC2992463

[52] Palmer SK, Zamudio S, Coffin C Quantitative estimation of human uterine artery blood flow and pelvic blood flow redistribution in pregnancy. Obstetr Gynecol 1992;80:1000–6. 1448242

[53] Palmer SK, Moore LG, Young DA Altered blood pressure course during normal pregnancy and increased preeclampsia at high altitude (3100 meters) in Colorado. Am J Obstet Gynecol 1999;180:1161–8. 10329872 10.1016/s0002-9378(99)70611-3

[54] Moore LG, Zamudio S, Zhuang J Oxygen Transport in Tibetan Women During Pregnancy at 3,658 m. Am J Phys Anthropol 2001;114:42–53. 11150051 10.1002/1096-8644(200101)114:1<42::AID-AJPA1004>3.0.CO;2-B

[55] Vargas M, Vargas E, Julian CG Determinants of blood oxygenation during pregnancy in Andean and European residents of high altitude. Am J Physiol Regul Integr Comp Physiol 2007;293:R1303–12. 17609312 10.1152/ajpregu.00805.2006

[56] Gonzales GF, Tapia V, Fort AL. Maternal and perinatal outcomes in second hemoglobin measurement in nonanemic women at first booking: effect of altitude of residence in Peru. ISRN Obstetr Gynecol 2012;2012. 10.5402/2012/368571 PMC3345214 22577573

[57] Gonzales GF, Tapia V, Gasco M Maternal hemoglobin concentration and adverse pregnancy outcomes at low and moderate altitudes in Peru. J Matern Fetal Neonatal Med 2012;25:1105–10. 22004716 10.3109/14767058.2011.623200

[58] Maghsoudlou S, Cnattingius S, Stephansson O Maternal haemoglobin concentrations before and during pregnancy and stillbirth risk: a population-based case-control study. BMC Pregnancy Childbirth 2016;16:135. 27259282 10.1186/s12884-016-0924-x PMC4893297

[59] Wang X, Byars SG, Stearns SC. Genetic links between post-reproductive lifespan and family size in Framingham. Evol Med Public Health 2014;2013:241–53. 10.1093/emph/eot013 PMC3868361 24481203

[60] Byars SG, Ewbank D, Govindaraju DR Colloquium papers: Natural selection in a contemporary human population. Proc Natl Acad Sci 2010; 107 (Suppl. 1):1787–92. 19858476 10.1073/pnas.0906199106 PMC2868295

[61] Stefansson H, Helgason A, Thorleifsson G A common inversion under selection in Europeans. Nat Genet 2005;37:129–37. 15654335 10.1038/ng1508

[62] Stearns SC, Byars SG, Govindaraju DR Measuring selection in contemporary human populations. Nat Rev Genet 2010;11:611–22. 20680024 10.1038/nrg2831

[63] Courtiol A, Pettay JE, Jokela M Natural and sexual selection in a monogamous historical human population. Proc Natl Acad Sci USA 2012;109:8044–9. 22547810 10.1073/pnas.1118174109 PMC3361384

[64] Kirk KM, Blomberg SP, Duffy DL Natural selection and quantitative genetics of life-history traits in Western women: a twin study. Evolution 2001;55:423–35. 11308097 10.1111/j.0014-3820.2001.tb01304.x

[65] Beauchamp JP. Genetic evidence for natural selection in humans in the contemporary United States. Proc Natl Acad Sci USA 2016;113:7774–9. 27402742 10.1073/pnas.1600398113 PMC4948342

[66] Kong A, Frigge ML, Thorleifsson G Selection against variants in the genome associated with educational attainment. Proc Natl Acad Sci USA 2017;114:E727–e732. 28096410 10.1073/pnas.1612113114 PMC5293043

[67] Tropf FC, Stulp G, Barban N Human fertility, molecular genetics, and natural selection in modern societies. PLoS One 2015;10:e0126821. 26039877 10.1371/journal.pone.0126821 PMC4454512

[68] Weeden J, Abrams MJ, Green MC Do high-status people really have fewer children?: Education, income, and fertility in the contemporary U.S. Hum Nat 2006;17:377–92. 26181608 10.1007/s12110-006-1001-3

[69] Stulp G, Sear R, Schaffnit SB The reproductive ecology of industrial societies, Part II: the association between wealth and fertility. Hum Nat 2016;27:445–70. 27670437 10.1007/s12110-016-9272-9 PMC5107208

[70] Stulp G, Barrett L, Tropf FC Does natural selection favour taller stature among the tallest people on earth?. Proc Biol Sci 2015;282:20150211 25854890 10.1098/rspb.2015.0211 PMC4426629

