# Supplementary material for: Ethnically Tibetan women in Nepal with low hemoglobin concentration have better reproductive outcomes
Source: Evol Med Public Health. 2017 Apr 21;2017(1):82–96. doi: 10.1093/emph/eox008 (PMC5442430; doi:10.1093/emph/eox008)
Supplement: Supplementary Data [file eox008_Supp.zip › USE Supplemental Table 5 revised.docx]

## Supplemental Table 5. Descriptive Statistics for variables

### 5.a. Continuous Variables

|  | **mean (SD)** | **median** | **# missing** |
| --- | --- | --- | --- |
| **Dependent Variables** | | | |
| Pregnancies | 5.68 (2.89) | 6 |  |
| Live Births | 5.35 (2.77) | 5 |  |
| Survival to age 15 | 3.96 (2.14) | 4 |  |
| **Independent Variables** | | | |
| Mother age at last birthpregnancy | 36.22 (7.91) | 37 | 4 (0.4%) |
| Mother age at first birth | 24.09 (4.61) | 23 | 10 (1.0%) |
| Age | 54.81 (10.74) | 54 |  |
| Altitude of residence | 3628.84 (243.94) | 3632 |  |
| Hb (gm/dL) | 13.84 (1.51) | 13.8 | 56 (5.6%) |
| Sat (%) | 87.35 (4.60) | 88 | 56 (5.6%) |
| Pulse (f/min) | 73.96 (10.89) | 73 | 58 (5.8%) |

### 5.b. Categorical Variables

| **Dependent variables** | | **Count (%)** | **# missing** |
| --- | --- | --- | --- |
| # Pregnancies | 0 | 20 (2.0) |  |
|  | 1 | 68 (6.8) |  |
|  | 2 | 62 (6.2) |  |
|  | 3 | 95 (9.4) |  |
|  | 4 | 118 (11.7) |  |
|  | 5 | 117 (11.6) |  |
|  | 6 | 125 (12.4) |  |
|  | 7 | 132 (13.1) |  |
|  | 8 | 99 (9.8) |  |
|  | 9 | 72 (7.2) |  |
|  | 10 | 49 (4.9) |  |
|  | 11 | 27 (2.7) |  |
|  | 12 | 11 (1.1) |  |
|  | 13 | 8 (0.8) |  |
|  | 14 | 2 (0.2) |  |
|  | 15 | 1 (0.1) |  |

| # Live Births | 0 | 26 (2.6) |  | |
| --- | --- | --- | --- | --- |
|  | 1 | 73 (7.3) |  |  |
|  | 2 | 76 (7.6) |  |  |
|  | 3 | 94 (9.3) |  |  |
|  | 4 | 123 (12.2) |  |  |
|  | 5 | 133 (13.2) |  |  |
|  | 6 | 128 (12.7) |  |  |
|  | 7 | 125 (12.4) |  |  |
|  | 8 | 93 (9.2) |  |  |
|  | 9 | 68 (6.8) |  |  |
|  | 10 | 34 (3.4) |  |  |
|  | 11 | 18 (1.8) |  |  |
|  | 12 | 11 (1.1) |  |  |
|  | 13 | 3 (0.3) |  |  |
|  | 14 | 1 (0.1) |  |  |
| # Livebirths that survived to age 15 | 0 | 17 (3.10) | | 1 (0.18) |
|  | 1 | 76 (13.87) | |  |
|  | 2 | 75 (13.69) | |  |
|  | 3 | 89 (16.24) | |  |
|  | 4 | 90 (16.42) | |  |
|  | 5 | 96 (17.52) | |  |
|  | 6 | 55 (10.04) | |  |
|  | 7 | 31 (5.66) | |  |
|  | 8 | 14 (2.56) | |  |
|  | 9 | 2 (0.37) | |  |
|  | 10 | 2 (0.37) | |  |

| **Independent Variables** | | | |
| --- | --- | --- | --- |
| **Direct determinants of exposure to intercourse** | | | |
| Current marital status | 1=married  2=widowed  3=divorced/separated  4=never married | 659 (65.51)  252 (25.05)  40 (3.98)  55 (5.47) |  |
| Continuously married (one marriage throughout ages 25-40) |  |  |  |
|  | 0 = no  1=yes | 405 (40.26)  601 (59.74) |  |
| **Direct determinants of susceptibility to conception and successful gestation** | | | |
| Use of contraception | 0 = never | 682 (67.79) | 10 (0.99) |
|  | 1 = past | 147 (14.61) |  |
|  | 2 = now | 167 (16.6) |  |
| # of twin pregnancies | 0  1  2 | 963(95.7)  41 (4.10)  2 (0.20) |  |
| # of miscarriages | 0  1  2  3 | 878 (87.30)  95 (9.40)  30 (3.00)  3 (0.3) |  |
| # of stillbirths | 0  1  2  3  4-6 | 886 (88.1)  98 (9.70)  14 (1.40)  4 (0.40)  4 (0.40) |  |
| **Indirect determinants of reproductive success** | | | |
| District of residence | 1 = Gorkha | 264 (26.24) |  |
|  | 2 = Mustang | 742 (73.76) |  |
| Sub-district of residence | 1 = Nubri | 132 (13.12) |  |
|  | 2 = Tsum | 132 (13.12) |  |
|  | 3 = Baragaon | 137 (13.62) |  |
|  | 4 = Upper Mustang | 605 (60.14) |  |
| Type of marriage | 0 = never married | 55 (5.47) |  |
|  | 1 = married to cousin, not polyandrous | 72 (7.16) |  |
|  | 2 = married not to cousin, is polyandrous | 94 (9.34) |  |
|  | 3 = married to cousin and polyandrous | 16 (1.59) |  |
|  | 4 = married not to cousin, not polyandrous | 769 (76.44) |  |
| Relative wealth rank | 1 = wealthy | 148 (14.71) | 83 (8.25) |
|  | 2 | 248 (24.65) |  |
|  | 3=middle  4  5 = poor | 209 (20.78) |  |
| Residence of first marriage | 0=never married  1=woman moved to husband’s home  2=man moved to wife’s home  3=couple made a new household | 57 (5.67)  728 (72.37)  118 (11.73)  103 (10.24) |  |
| Education status | 0 = no education for either | 437 (43.44) |  |
|  | 1 = no education wife, some for husband | 522 (51.89) |  |
|  | 2 = some education for wife, none for husband | 16 (1.59) |  |
|  | 3 = some education for both | 31 (3.08) |  |
| Living in natal village | 0 = no | 423 (42.05) | 11 (1.09) |
|  | 1 = yes | 572 (56.86) |  |
|  |  |  |  |
|  |  |  |  |
