# Supplementary material for: Ethnically Tibetan women in Nepal with low hemoglobin concentration have better reproductive outcomes
Source: Evol Med Public Health. 2017 Apr 21;2017(1):82–96. doi: 10.1093/emph/eox008 (PMC5442430; doi:10.1093/emph/eox008)
Supplement: Supplementary Data [file eox008_Supp.zip › 2nd revision Supplemental Table 6.docx]

## Supplemental Table 6. Quasi-R^2^ calculations for reported analyses

|  | **Null Deviance** | **N.D. df** | **Residual Deviance** | **R.D. df** | **Quasi-R^2^** | **numerator** | **denominator** | **Adjusted Quasi-R^2^** |
| --- | --- | --- | --- | --- | --- | --- | --- | --- |
| WHOLE SAMPLE |  |  |  |  |  |  |  |  |
| # pregnancies | 1255.27 | 862 | 321.61 | 845 | 0.74379217 | 0.38060355 | 1.4562297 | 0.7386377 |
| # Live births | 1429.57 | 981 | 53.32 | 971 | 0.96270207 | 0.05491246 | 1.4572579 | 0.96231795 |
| # survive to age 15 | 675.74 | 541 | 257.94 | 521 | 0.61828514 | 0.49508637 | 1.2490573 | 0.60363198 |
