# Supplementary material for: Ethnically Tibetan women in Nepal with low hemoglobin concentration have better reproductive outcomes
Source: Evol Med Public Health. 2017 Apr 21;2017(1):82–96. doi: 10.1093/emph/eox008 (PMC5442430; doi:10.1093/emph/eox008)
Supplement: Supplementary Data [file eox008_Supp.zip › USE Supplemental Table 1 revised.docx]

## Supplemental Table 1. Reasons for exclusion or non-participation

| Reason | Gorkha District, # Women | Mustang District, # Women |
| --- | --- | --- |
| Total participant pool | 334 | 942 |
| Participated and excluded from final sample | 6 | 18 |
| Non-participants |  |  |
| - Birthplace < 3000m | 0 | 3 |
| - Celibate nun | 17 | 4 |
| - Disability | 3 | 0 |
| - Away from home | 2 | 17 |
| - Never married or pregnant | 21 | 33 |
| - Total excluded by selection criteria | 43 | 57 |
| - Other or unknown reason for exclusion or non-participation | 32 (9.5%) | 76 (8.1%) |
