# Supplementary material for: Ethnically Tibetan women in Nepal with low hemoglobin concentration have better reproductive outcomes
Source: Evol Med Public Health. 2017 Apr 21;2017(1):82–96. doi: 10.1093/emph/eox008 (PMC5442430; doi:10.1093/emph/eox008)
Supplement: Supplementary Data [file eox008_Supp.zip › USE Supplemental Table 2 revised.docx]

## Supplemental Table 2. Residence altitude of the sample of Tibetan women

| Altitude | N of women , total n=1006 | % of total sample | % Living in their natal village |
| --- | --- | --- | --- |
| 3000-3099 | 30 | 3 | 50 |
| 3100-3199 | 49 | 4.9 | 82 |
| 3200-3299 | 69 | 6.9 | 42 |
| 3300-3399 | 31 | 3.1 | 67 |
| 3400-3499 | 35 | 3.5 | 51 |
| 3500-3599 | 279 | 27.7 | 73 |
| 3600-3699 | 37 | 3.7 | 8 |
| 3700-3799 | 192 | 19.1 | 72 |
| 3800-3899 | 155 | 15.4 | 23 |
| 3900-3999 | 113 | 11.2 | 63 |
| 4000-4099 | 16 | 1.6 | 0 |

The mean barometric pressure was 501 mmHg with a temperature of 17 °C (63 °F) and relative humidity of 35%, based on measurements obtained between 6 and 7 am.
