# Supplementary material for: Ethnically Tibetan women in Nepal with low hemoglobin concentration have better reproductive outcomes
Source: Evol Med Public Health. 2017 Apr 21;2017(1):82–96. doi: 10.1093/emph/eox008 (PMC5442430; doi:10.1093/emph/eox008)
Supplement: Supplementary Data [file eox008_Supp.zip › USE Supplemental Table 3 revised.docx]

## Supplemental Table 3. Repeatability of biological measurements among 51 Tibetan women

| Measurement | First measurement  (mean + SD) | Second Measurement  (mean + SD) | Average difference  (time 2 – time 1) | Intraclass correlation (ICC) |
| --- | --- | --- | --- | --- |
| Hemoglobin concentration, gm/dL | 14.5 + 1.32 | 14.3 + 1.30 | -.25 + 1.31 | 0.66 |
| %Oxygen saturation | 88.9 + 3.94 | 87.6 + 4.64 | -1.3 + 3.31 | 0.81 |
| Pulse, f/minute | 72.8 + 10.35 | 73.7 + 11.63 | + 1.1 + 11.32 | 0.65 |

The analysis does not include two women with hemoglobin concentrations well below the normal sea level range at their first measurement who had been referred to nearby health facilities for treatment to raise their levels.
