# Supplementary material for: Ethnically Tibetan women in Nepal with low hemoglobin concentration have better reproductive outcomes
Source: Evol Med Public Health. 2017 Apr 21;2017(1):82–96. doi: 10.1093/emph/eox008 (PMC5442430; doi:10.1093/emph/eox008)
Supplement: Supplementary Data [file eox008_Supp.zip › USE Supplemental Table 4 revised.docx]

## Supplemental Table 4. Variable list

| **Dependent Variables** |  | | |
| --- | --- | --- | --- |
| Pregnancies |  | | |
| Live Births |  | | |
| Survival to age 15 |  | | |
| **Independent Variables** | |  |  |
| **Direct determinants of exposure to intercourse** | | |  |
| Current marital status | |  |  |
| Continuously married (one marriage throughout ages 25-40) | | |  |
| **Direct determinants of susceptibility to conception** | | |  |
| Use of contraception | |  |  |
| # of twin pregnancies | |  |  |
| # of miscarriages | |  |  |
| # of stillbirths | |  |  |
| Age at last pregnancy/birth | |  |  |
| Age at first pregnancy/ birth | |  |  |
| Age in Western years | |  |  |
| **Indirect determinants of reproductive success** | | |  |
| District of residence | |  |  |
| Sub-district of residence | |  |  |
| Type of marriage | |  |  |
| Household relative wealth rank | |  |  |
| Residence of first marriage | | |  |
| Education status of woman and husband education | |  |  |
| Living in natal village | |  |  |
| Altitude of residence | |  |  |
| **Physiological Phenotypes** | |  |  |
| Hemoglobin concentration (gm/dL) | |  |  |
| Percent of oxygen saturation of hemoglobin (%)Sat (%) | |  |  |
| Pulse (f/min) | |  |  |
